# Personality and incident disabling dementia among community-dwelling older adults: a 20-year longitudinal study

**DOI:** 10.1093/geroni/igaf122.2923

**Published:** 2025-12-31

**Authors:** Yukiko Nishita, Chikako Tange, Shu Zhang, Sayaka Kubota, Mana Tateishi, Fujiko Ando, Hiroshi Shimokata, Rei Otsuka

**Affiliations:** National Center for Geriatrics and Gerontology, Obu, Aichi, Japan; National Center for Geriatrics and Gerontology, Obu, Aichi, Japan; National Center for Geriatrics and Gerontology, Obu, Aichi, Japan; National Center for Geriatrics and Gerontology, Obu, Aichi, Japan; National Center for Geriatrics and Gerontology, Obu, Aichi, Japan; Aichi Shukutoku University, Nagakute, Aichi, Japan; Nagoya University of Arts and Sciences, Nisshin, Aichi, Japan; National Center for Geriatrics and Gerontology, Obu, Aichi, Japan

## Abstract

**Objectives:**

This study examines the effect of personality on incident disabling dementia in community-dwelling older adults.

**Methods:**

We analyzed 898 adults (65 − 84 years) from the National Institute for Longevity Sciences-Longitudinal Study of Aging 2nd (2000 − 2002) or 5th (2006 − 2008) waves, with baseline established at their first personality questionnaire response. Participants with baseline cognitive impairment (MMSE ≤ 23), incident disabling dementia before baseline or within five follow-up years were excluded. Personality was assessed using NEO Five-Factor Inventory, measuring neuroticism, extraversion, openness, agreeableness, and conscientiousness. Disabling dementia was defined as ≥ rank II on the “Independence Degree in Daily Living for Older adults with Dementia” based on Doctor’s Opinion Paper (from baseline to January 2022). Cox proportional hazards models estimated adjusted hazard ratios (aHR) and 95% confidence intervals (CI) for one standard deviation (SD) increase of personalities, controlling for age, sex, education, hearing and vision impairment, LDL cholesterol, depressive symptoms, physical activity, history of diabetes and hypertension, smoking, alcohol drinking, body mass index, social isolation, APOE ε4, and survey wave. A stratified analysis of APOE ε4 was also conducted.

**Results:**

Over a mean (SD) follow-up of 12.3 (5.8) years, 196 individuals (21.8%) developed disabling dementia. Openness was negatively associated with overall incident disabling dementia risk (aHR [95%CI]: 0.82 [0.70–0.96]). Stratified analysis showed that openness reduced dementia risk in ε4 non-carriers (0.79 [0.67–0.94]), while conscientiousness reduced it in ε4 carriers (0.56 [0.37–0.84]).

**Conclusion:**

Openness may reduce incident disabling dementia risk, while conscientiousness might suppress disabling dementia onset in ε4 carriers.

